# Supplementary material for: A systematic review of the methodological considerations in Campylobacter burden of disease studies
Source: PLoS Negl Trop Dis. 2025 Apr 22;19(4):e0012681. doi: 10.1371/journal.pntd.0012681 (PMC12013896; doi:10.1371/journal.pntd.0012681)
Supplement: S2 File — (PDF) [file pntd.0012681.s002.pdf]

S2 File. Search Strategy for Study Inclusion

S2 Table 1. Search Strategy for PubMed

**Number**	**Search Term**	**Search Query**	**Articles Retrieved**
1	Campylobacter	"Campylobacter" [Mesh]	12,968
2	Campylobacteraceae	Campylobacteraceae	13,055
3	Burden of Disease	"Burden of disease"	15,358
4	Disability-adjusted Life Year	"Disability-adjusted life year"	1,058
5	Disability-adjusted Life Years	"Disability-adjusted life years"	4,458
6	DALY	DALY	16,360
7	DALYs	DALYs	5,935
8	Years of Life Lost	"Years of life lost"	2,354
9	Years Lived with Disability	"Years lived with disability"	1,094
10	YLL	YLL	976
11	YLD	YLD	588
12	Cost Effectiveness	"Cost effectiveness"	75,425
13	Cost of Illness	"Cost of illness"	32,799
14	Campylobacter (Combined)	("Campylobacter"[Mesh]) OR (Campylobacteraceae)	13,055
15	Burden & Disability (Combined)	(((((((((("Burden of disease") OR ("Disability-adjusted life year")) OR ("Disability-adjusted life years")) OR (DALY)) OR (DALYs)) OR ("Years of life lost")) OR ("Years lived with disability")) OR (YLL)) OR (YLD)) OR ("Cost effectiveness")) OR ("Cost of illness")	136,425

16	Focused Query	((“Campylobacter”[Mesh]) OR (Campylobacteraceae)) AND ((((((((((“Burden of disease”) OR (“Disability-adjusted life year”)) OR (“Disability-adjusted life years”)) OR (DALY)) OR (DALYs)) OR (“Years of life lost”)) OR (“Years lived with disability”)) OR (YLL)) OR (YLD)) OR (“Cost effectiveness”)) OR (“Cost of illness”))	52
17	Final Query with Filters	((“Campylobacter”[Mesh]) OR (Campylobacteraceae)) AND ((((((((((“Burden of disease”) OR (“Disability-adjusted life year”)) OR (“Disability-adjusted life years”)) OR (DALY)) OR (DALYs)) OR (“Years of life lost”)) OR (“Years lived with disability”)) OR (YLL)) OR (YLD)) OR (“Cost effectiveness”)) OR (“Cost of illness”)) Filters: Abstract; Species: Humans; Year: 1990-2023	49

S2 Table 2. Search Strategy for Embase

Number	Search Term	Search Query	Articles Retrieved
1	Campylobacter	Campylobacter	26,604
2	Campylobacteraceae	Campylobacteraceae	209
3	Burden of Disease	‘Burden of disease’	20,743
4	Disability-adjusted Life Year	‘Disability-adjusted life year’	4,993
5	Disability-adjusted Life Years	‘Disability-adjusted life years’	5,289
6	DALY	DALY	21,191
7	DALYs	DALYs	4,318
8	Years of Life Lost	‘Years of life lost’	2,931
9	Years lived with Disability	‘Years lived with disability’	1,385
10	YLL	YLL	1,370
11	YLD	YLD	755
12	Cost Effectiveness	‘Cost effectiveness’	210,195
13	Cost of Illness	‘Cost of illness’	22,680
14	Combined Campylobacter Terms	#1 OR #2	26,745
15	Combined Burden and Disability Terms	#3 #4 OR #5 OR #6 OR #7 OR #8 OR #9 OR #10 OR #11 OR #12 OR #13	272,159
16	Combined Query	#14 AND #15	246
17	Final Query with Filters	#14 AND #15 AND [1966-2023]/py AND [humans]/lim	191

S2 Table 3. Search Strategy for Web of Science

Number	Search Term	Search Query	Articles Retrieved
1	Campylobacter	TS=(Campylobacter)	29,187
2	Campylobacteraceae	TS=(Campylobacteraceae)	104
3	Combined Campylobacter Terms	#1 OR #2	29,238
4	Burden of Disease	TS=("Burden of disease")	15,403
5	Disability-adjusted Life Year	TS=("Disability-adjusted life year")	5,254
6	Disability-adjusted Life Years	TS=("Disability-adjusted life years")	5,254
7	DALY	TS=DALY	4,160
8	DALYs	TS=DALYs	3,494
9	Years of Life Lost	TS=("Years of life lost")	2,232
10	Years Lived with Disability	TS=("Years lived with disability")	1,039
11	YLL	TS=(YLL)	769
12	YLD	TS=(YLD)	591
13	Cost Effectiveness	TS=("Cost effectiveness")	126,701
14	Cost of Illness	TS=("Cost of illness")	3,994
15	Combined Burden and Cost Terms	#4 OR #5 OR #6 OR #7 OR #8 OR #9 OR #10 OR #11 OR #12 OR #13 OR #14	150,271
16	Combined Campylobacter and Burden/Cost Terms	(#3 AND #15)	152
17	Final Query with Filters	(#3 AND #15) AND ALL=(Humans)	40

S2 Table 4. Search Strategy for Grey Literature: OAIster

Number	Search Term	Search Query	Articles Retrieved	Notes
1	Campylobacter and Related Terms	kw:(Campylobacter) OR ti:(Campylobacteraceae) AND ti:("Burden of disease") OR ti:("Disability-adjusted life year") OR ti:("Disability-adjusted life years") OR ti:(DALY) OR ti:(DALYs) OR ti:("Years of life lost") OR ti:("Years lived with disability") OR ti:(YLL) OR ti:(YLD) OR ti:("Cost effectiveness") OR ti:("Cost of illness")	15,000	
2	Campylobacter and Related Terms (Filtered)	kw:(Campylobacter) OR ti:(Campylobacteraceae) AND ti:("Burden of disease") OR ti:("Disability-adjusted life year") OR ti:("Disability-adjusted life years") OR ti:(DALY) OR ti:(DALYs) OR ti:("Years of life lost") OR ti:("Years lived with disability") OR ti:(YLL) OR ti:(YLD) OR ti:("Cost effectiveness") OR ti:("Cost of illness") NOT ti:(Cancer)	14,200	**Filtered Results:**<br>- Excluded: Cancer<br>- Total Articles Returned: 14,200<br>- Articles Searched: First 100<br>- Articles Included: 1

S2 Table 5. Search Strategy for Grey Literature: Google Scholar

Number	Search Term	Search Query	Articles Retrieved	Notes
1	Campylobacter and Disability	“Campylobacter” AND “disability-adjusted life year”	867	**Search parameters:**<br>- All words: Campylobacter AND "disability-adjusted life years"<br>- Occurrence: Anywhere in the article<br>- Date Range: 1990-2023<br><br>**Results Overview:**<br>- Total Articles Returned: 867<br>- Articles Searched: First 100<br>- Articles Included: 8
