# Supplementary material for: A systematic review of the methodological considerations in Campylobacter burden of disease studies
Source: PLoS Negl Trop Dis. 2025 Apr 22;19(4):e0012681. doi: 10.1371/journal.pntd.0012681 (PMC12013896; doi:10.1371/journal.pntd.0012681)
Supplement: S4 File — (PDF) [file pntd.0012681.s004.pdf]

S4 File. Campylobacteriosis Disease Models

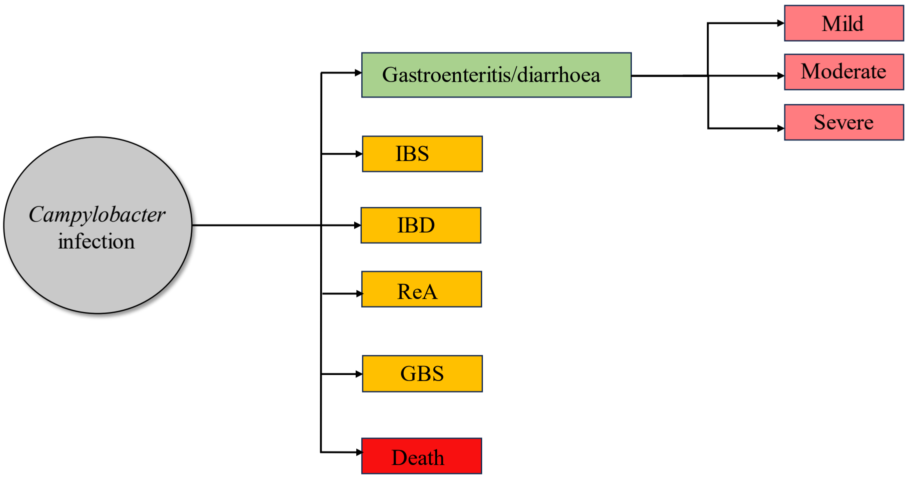

*IBS = irritable bowel syndrome; IBD = inflammatory bowel disease;ReA = reactive arthritis; GBS = Guillain-Barré syndrome

S4 Fig 1. Disease model 1

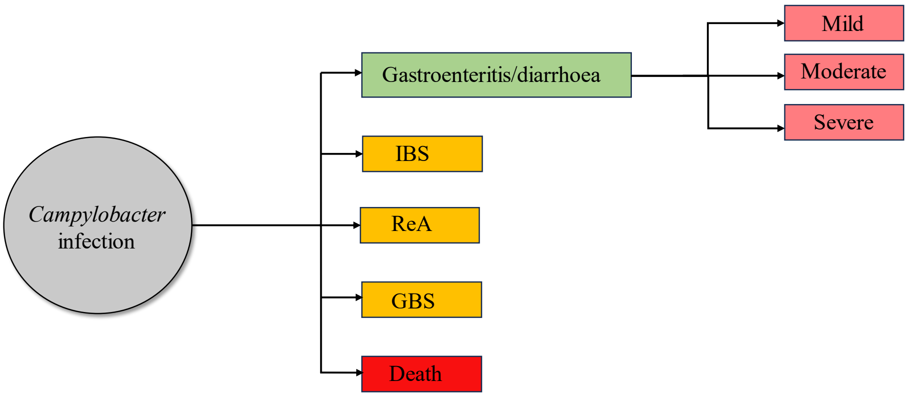

*IBS = irritable bowel syndrome;ReA = reactive arthritis; GBS = Guillain-Barré syndrome

S4 Fig 2. Disease model 2

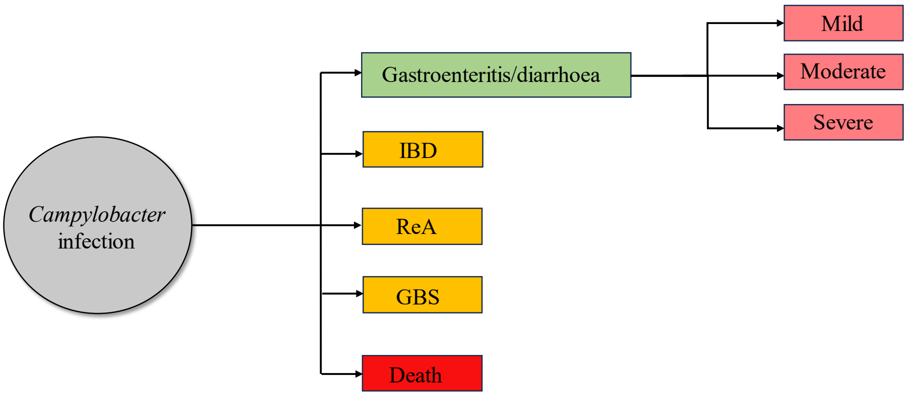

*IBD = inflammatory bowel disease; ReA = reactive arthritis; GBS = Guillain-Barré syndrome

S4 Fig 3. Disease model 3

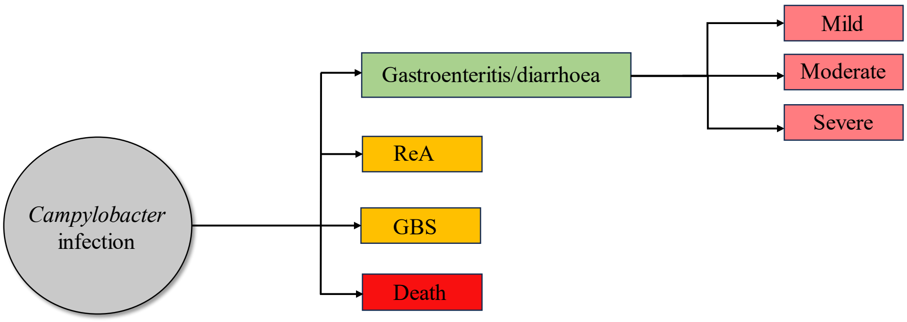

*ReA = reactive arthritis; GBS = Guillain-Barré syndrome

S4 Fig 4. Disease model 4

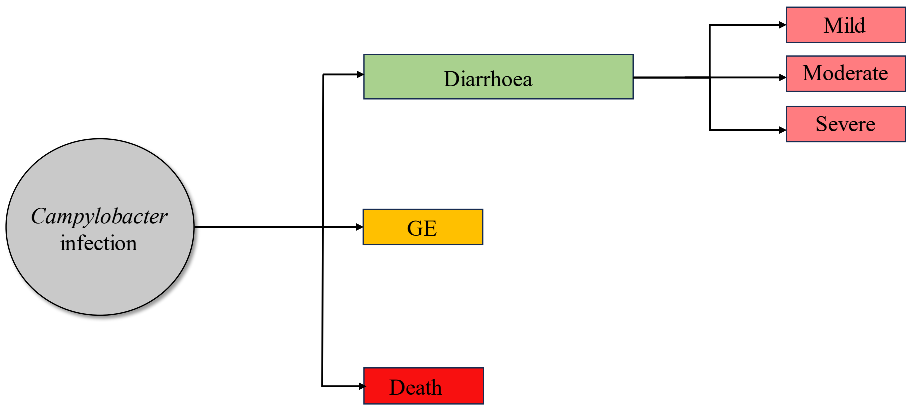

*GE = gastroenteritis

S4 Fig 5. Disease model 5

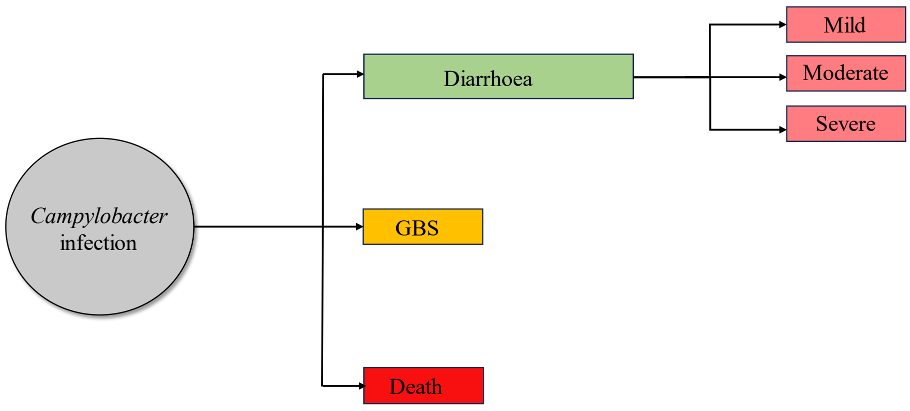

*GBS = Guillain-Barré syndrome

S4 Fig 6. Disease model 6

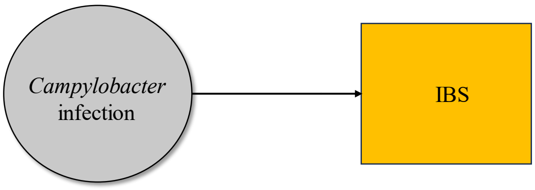

*IBS = irritable bowel syndrome

S4 Fig 7. Disease model 7
