# Supplementary material for: A systematic review of the methodological considerations in Campylobacter burden of disease studies
Source: PLoS Negl Trop Dis. 2025 Apr 22;19(4):e0012681. doi: 10.1371/journal.pntd.0012681 (PMC12013896; doi:10.1371/journal.pntd.0012681)
Supplement: S5 File — (PDF) [file pntd.0012681.s005.pdf]

S5 File. Methodology for Calculating DALYs

The Disability-Adjusted Life Years (DALYs) estimation "*simultaneously recognises the decreased health state due to disability prior to mortality and the reduction in life expectancy due to mortality*" (1). DALYs combine years of life lost due to premature mortality (YLL) and years lived with disability (YLD) into a single standardized metric (2–4). The formula used to calculate DALYs is:

$$DALY = YLL + YLD$$

This formula quantifies these components, where YLL considers premature deaths adjusted for residual life expectancy and age at death (5,6). The YLD component can be computed from either an incidence or prevalence perspective. Following an incidence perspective, YLDs for a given health state are defined as the number of incident cases, the duration until remission or mortality, and the disability weight (DW) (7). This perspective, adopted by the BCoDE and WHO/FERG studies, forecasts future BoD due to current exposures (3,7,8). Moreover, both the BCoDE and WHO/FERG studies follow a pathogen-based approach, examining predominant outcomes associated with a particular pathogen, including sequelae (8).

$$YLD = incidence\ x\ duration\ x\ DW$$

In comparison, the prevalence perspective, utilised by the GBD study, captures the health status of a population at a specific moment, calculated as the product of the number of prevalent cases and the associated DW (3,5,7). Additionally, the GBD study adopts an

outcome-based approach, distributing the BoD among categorised health conditions and providing estimates for major infectious disease-related outcomes (8).

$$YLD = prevalence\ x\ DW$$

DWs, crucial for both perspectives, are weighting factors that gauge the severity of a health state and the corresponding decline in quality of life, ranging from 0 (full health) to 1 (death) (7,8). Additionally, the selection of appropriate life expectancy tables is pivotal in DALY calculations, translating age-specific mortality rates into estimates for calculating YLLs (9). DALY calculations may also incorporate age weighting or time discounting to reflect values at different ages or time periods, respectively (10). However, the adoption of these social-weighting functions is often debated in the literature, with declining acceptance among researchers (10).

References

1. Kim YE, Jung YS, Ock M, Yoon SJ. DALY Estimation Approaches: Understanding and Using the Incidence-based Approach and the Prevalence-based Approach. J Prev Med Pub Health. 2022 Jan;55(1):10–8.

2. Cuschieri S, Alkerwi A, Economou M, Idavain J, Lai T, Lesnik T, et al. Conducting national burden of disease studies and knowledge translation in eight small European states: challenges and opportunities. Health Res Policy Syst. 2022 Oct 21;20(1):113.

3. Di Bari C, Venkateswaran N, Bruce M, Fastl C, Huntington B, Patterson GT, et al. Methodological choices in brucellosis burden of disease assessments: A systematic review. PLoS Negl Trop Dis. 2022 Dec 13;16(12):e0010468.

4. Murray CJ. Quantifying the burden of disease: the technical basis for disability-adjusted life years. Bull World Health Organ. 1994;72(3):429–45.

5. Devleesschauwer B, Bouwknegt M, Mangen MJJ, Havelaar AH. Chapter 2 - Health and economic burden of Campylobacter. In: Klein G, editor. Campylobacter: Features, Detection, and Prevention of Foodborne Disease. Hannover, Germany: Academic Press; 2017. p. 27–40.

6. World Health Organisation. The Global Health Observatory. 2023 [cited 2023 May 19]. Years of life lost from mortality (YLL). Available from: https://www.who.int/data/gho/indicator-metadata-registry/imr-details/159

7. Devleesschauwer B, Haagsma JA, Angulo FJ, Bellinger DC, Cole D, Döpfer D, et al. Methodological framework for World Health Organization estimates of the global burden of foodborne disease. PLoS ONE. 2015;10(12).

8. Charalampous P, Polinder S, Wothge J, von der Lippe E, Haagsma JA. A systematic literature review of disability weights measurement studies: evolution of methodological choices. Arch Public Health. 2022 Mar 24;80(1):91.

9. von der Lippe E, Devleesschauwer B, Gourley M, Haagsma J, Hilderink H, Porst M, et al. Reflections on key methodological decisions in national burden of disease assessments. Arch Public Health. 2020 Dec 31;78(1):137.

10. Devleesschauwer B, Havelaar AH, Maertens de Noordhout C, Haagsma JA, Praet N, Dorny P, et al. Calculating disability-adjusted life years to quantify burden of disease. Int J Public Health. 2014 Jun 1;59(3):565–9.
