# Supplementary material for: A systematic review of the methodological considerations in Campylobacter burden of disease studies
Source: PLoS Negl Trop Dis. 2025 Apr 22;19(4):e0012681. doi: 10.1371/journal.pntd.0012681 (PMC12013896; doi:10.1371/journal.pntd.0012681)
Supplement: S6 File — (PDF) [file pntd.0012681.s006.pdf]

S6 File. DALYs Per Case Calculations

The DALYs per case were calculated by dividing the total DALY estimate by the number of reported cases, where such data was available in the studies. If the number of cases was not reported in the study, it was not possible to estimate DALYs per case.

For studies that reported DALY estimates for multiple years, DALYs per case were calculated separately for each individual year. Specifically, the total DALYs for each year were divided by the corresponding number of reported cases. This approach ensured that the most precise and accurate data available was used for each year within the study period.

For example, in the study by van Lier *et al.* (2016) (1), the estimated annual DALYs for campylobacteriosis were 3,114 DALYs for the period from 2007 to 2011. To calculate DALYs per case for each year within this period, the following method was applied:

- In 2007, with 6,731 reported cases for campylobacteriosis, DALYs per case were calculated as 3,114 ÷ 6,731 = 0.492 DALYs per case.
- This calculation was then repeated for each subsequent year, using the corresponding case numbers (e.g., 6,431 cases in 2008).

References

1. van Lier A, McDonald SA, Bouwknegt M, Group EPI, Kretzschmar ME, Havelaar AH, et al. Disease Burden of 32 Infectious Diseases in the Netherlands, 2007-2011. PLOS ONE. 2016 Apr 20;11(4):e0153106.
